# Reduced *cul-5* Activity Causes Aberrant Follicular Morphogenesis and Germ Cell Loss in *Drosophila* Oogenesis

**DOI:** 10.1371/journal.pone.0009048

**Published:** 2010-02-04

**Authors:** Jan-Michael Kugler, Christopher Lem, Paul Lasko

**Affiliations:** 1 Developmental Biology Research Initiative, Department of Biology, McGill University, Montréal, Québec, Canada; 2 Goodman Cancer Centre, McGill University, Montréal, Québec, Canada; Stockholm University, Sweden

## Abstract

*Drosophila* oogenesis is especially well suited for studying stem cell biology, cellular differentiation, and morphogenesis. The small modifier protein ubiquitin regulates many cellular pathways. Ubiquitin is conjugated to target proteins by a diverse class of enzymes called ubiquitin E3 ligases. Here we characterize the requirement of Cul-5, a key component of a subgroup of Cullin-RING-type ubiquitin E3 ligases, in *Drosophila* oogenesis. We find that reduced *cul-5* activity causes the formation of aberrant follicles that are characterized by excess germ cells. We show that germ line cells overproliferate in *cul-5* mutant females, causing the formation of abnormally large germ line cysts. Also, the follicular epithelium that normally encapsulates single germ line cysts develops aberrantly in *cul-5* mutant, leading to defects in cyst formation. We additionally found that Cul-5 is required for germ cell maintenance, as germ cells are depleted in a substantial fraction of *cul-5* mutant ovaries. All of these *cul-5* phenotypes are strongly enhanced by reduced activity of *gustavus (gus)*, which encodes a substrate receptor of Cul-5-based ubiquitin E3 ligases. Taken together, our results implicate Cul-5/Gus ubiquitin E3 ligases in ovarian tissue morphogenesis, germ cell proliferation and maintenance of the ovarian germ cell population.

## Introduction

Ubiquitination is a particularly versatile post-translational modification that influences most, if not all, aspects of cellular biology [1]. Conjugation of ubiquitin to a target protein occurs through an conserved enzymatic cascade that involves a ubiquitin activating enzyme (also called E1), a ubiquitin conjugating enzyme (E2) as well as a ubiquitin ligase (E3). E3 ligases confer substrate specificity to the ubiquitination reaction. In light of this crucial function, it is not surprising that many E3 ligases exist [1], [2], [3]. Among these, the Cullin RING E3 ligases (CRLs) are the largest family [4].

CRLs are modular protein complexes that are formed on a characteristic scaffold subunit of the Cullin protein family [2], [4]. The conserved C-terminal domain of the Cullin recruits a RING protein that enables the association of the CRL with an E2 conjugating enzyme. The N-terminal domain of the Cullin associates with a substrate receptor protein, which in turn recruits the target of the ubiquitination reaction. In some cases, adaptor proteins form a bridge between the Cullin and its substrate receptors. By bringing them in close proximity, the Cullin enables the transfer of ubiquitin from the E2 enzyme to the target protein. Notably, each Cullin can associate with several different substrate receptors, which moreover may recognize multiple targets. Therefore, the number of proteins regulated by a given Cullin can be quite large. Consequently, it is not surprising that Cullins have been implicated in many aspects of cellular biology, ranging from cell cycle progression, modulation of intra- and intercellular pathways, chromatin remodelling and many more.

Cullin-5 (Cul-5) has been most extensively studied in the context of viral infection [5], and has recently been implicated in the regulation of the non-receptor tyrosine kinase Src [6]. However, comparatively little is known about Cul-5 function in developmental and morphogenetic processes. In *Drosophila*, certain genetic manipulations of *cul-5* have been reported to affect epidermal patterning, synapse formation and sensory organ development [7]. In mice, Cul-5 is involved in neuron positioning during development of the brain cortex [8]. These findings indicate that Cul-5 is involved in various developmental and morphogenetic processes.

*Drosophila* oogenesis provides an excellent model system to study different aspects of cellular biology, intercellular communication and tissue morphogenesis [9], [10]. During oogenesis, mature eggs develop from multicellular progenitor structures called egg chambers. Egg chambers are produced in higher order structures called ovarioles in which progressively more mature egg chambers are lined up in a linear fashion. Each egg chamber contains a cyst (or cluster) of 16 interconnected germ cells (cystocytes) that arises from a common stem cell-derived progenitor (the cystoblast) through four rounds of synchronous mitotic divisions. Germ line stem cell (GSC) and subsequent cystocyte divisions take place in the most apical portion of the ovariole, in regions 1 and 2A of the germarium. Cystocyte divisions are incomplete, and the daughter cells remain connected by cytoplasmic bridges, called ring canals, that are stabilized as F-actin-rich structures. These allow the passage of material between cystocytes. Only one of the cyst cells enters meiosis and adopts an oocyte fate, while the other 15 endoreplicate and differentiate into nurse cells, which provide macromolecules and organelles to the developing oocyte.

In region 2B of the germarium, each germ line cyst becomes encapsulated by a monolayered follicular epithelium of somatic origin [9], [10], [11]. The follicular epithelium plays crucial roles in establishing and maintaining polarity of the egg chambers, and secretes the eggshell during late oogenesis before its own degeneration [11], [12]. Proper development of the follicular epithelium and consequently egg chamber morphogenesis requires communication between germ line and somatic cells as well as among follicle cells themselves, and involves among others the Notch/Delta and EGF receptor signalling pathways. In region 3 of the germarium, individual follicles bud off the germarium and enter the vitellarium.

Here, we demonstrate that reduced *cul-5* function leads to the formation of aberrantly formed follicles that are most frequently characterized by an excess of germ line cells. Their formation results both from germ cell overproliferation in the germarium, and improper clustering of germ cell cysts into inappropriately sized follicles. These phenotypes are drastically enhanced by sensitizing the *cul-5* mutant background by reducing genetic dosage of the Cul-5 CRL receptor Gustavus (Gus) [13] (JM.K. and P.L., submitted), indicating that Cul-5 acts at least in part through Gus to ensure normal ovarian morphogenesis.

Intriguingly, *cul-5* mutant ovarioles also display a partially penetrant germ cell depletion phenotype. Again, this phenotype is drastically enhanced by simultaneous reduction of the Cul-5 substrate receptor Gus. We therefore establish a novel functional link between a Cul-5 CRL and GSC maintenance or germ cell survival.

## Results

### *cul-5* Mutant Ovaries Contain Aberrantly Formed Follicles

**Figure 1:**
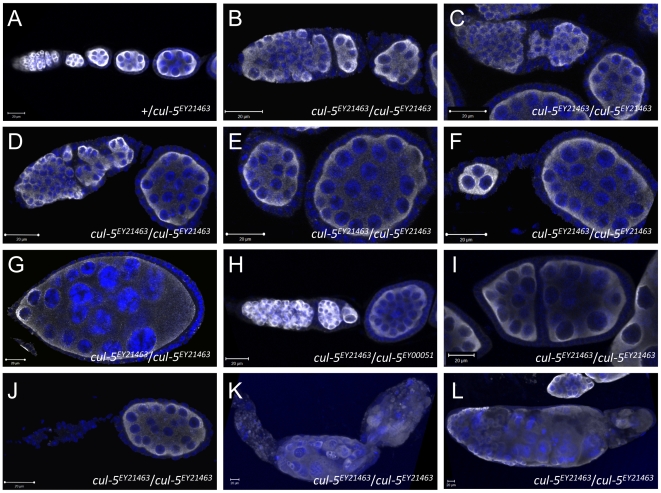
*cul-5* mutant ovarioles form aberrant egg chambers. Ovarioles from control (A) and *cul-5^EY21463^* mutant (B–L) females, labelled for Vas (white) and DNA (blue). (A, B) Control (A) and many *cul-5* mutant (B) ovarioles show normal morphology. In a substantial fraction of *cul-5* mutant ovarioles (C–J), one or several egg chambers contain more than 16 germ line cells (aberrant egg chambers). (C, D) Cysts in region III of *cul-5* mutant germaria are often irregularly shaped and fail to take up a oval or round shape as wild type cysts do. (E) Frequently, several follicles containing excess germ cells are observed in a single ovariole. (F) More rarely, *cul-5* mutant egg chambers contain less than 16 germ line cells. (G) More mature aberrant egg chambers can develop relatively normally. (H) Aberrant egg chambers are also observed in a different allelic combination. (I) In some *cul-5* mutant ovarioles, individual egg chambers are not separated by a stalk, but by two layers of follicular epithelium. (J) A fraction of *cul-5* ovarioles has germaria that do not contain germ cells. (K, L) In aged flies, a fraction of ovarioles undergoes morphogenetic catastrophe and lose the highly ordered structure of normally developing ovarioles. See Table 1 for quantification of this phenotype.

We noted that, unlike in wild type ovarioles (Fig. 1A; Table 1), a substantial fraction of follicles in females homozygous for the hypomorphic *cul-5^EY21463^* P-element insertion [14] contained obviously more than 16 germ line cells (Fig. 1B–1G). These additional cells show morphogenetic characteristics of germ cells, and are positive for two germ-cell specific markers, Vasa (Vas) (Fig. 1) and Aubergine (Aub) (not shown). In 34% of ovarioles isolated from 1–2d old *cul-5^EY21463^* mutant females (unless explicitly stated in the text, all experiments were performed with 1-2d old females) we observed at least one but often several follicle(s) containing an excess number of germ line cells (Fig. 1; Table 1). We also observed this (and other phenotypes discussed below) in ovaries that were either homozygous for the *cul-5^EY00051^* allele [7] (data not shown) or in *cul-5^EY00051^*/*cul-5^EY21463^* ovaries (Fig. 1H). These results suggest that wild type *cul-5* function is important for normal ovarian follicle morphogenesis.

**Table 1:** *cul-5* phenotypes as a function of age.

Age (days)	1–2	1–2	1–2	5–6	5–6	11–12	11–12	21–22	21–22
	OrR	*+/cul-5^EY21463^*	*cul-5^EY21463^*	OrR	*cul-5^EY21463^*	OrR	*cul-5^EY21463^*	OrR	*cul-5^EY21463^*
**n (ovarioles)**	**>111**	**111**	**105**	**119**	**141**	**199**	**173 (54)^5^**	**74**	**161**
no phenotype	100	98.2	65.7	100	36.9	99	34.7	98.6	43.5
≥1 tumorous follicle	0	1.8	34.3	0	61.7	0.5	57.8	0	37.3
others	0	0	0	0	1.4	0.5	3.5	1.4	6.2
morphogenetic catastrophe	0	0	0	0	0	0	11.1 (6)^5^	0	13
**additional phenotypes^1^**									
follicle with <16 germ cells^2^	0	0	7.6	0	11.1	0	6.9	0	7.5
follicle with <16 germ cells^3^	0	0	0	0	1.4	0	0	0	2.5
tumor split by somatic cells	0	0	3.8	0	4.2	0	1.7	0	3.7
empty germarium	0	0	2.9	0	9.7	0	18.5	0	9.9
others^4^	0	0	3.8	0	0.7	0	0	0	2.5

All numbers are percentages except for n, the number of ovarioles examined.

1 Unless otherwise indicated these phenotypes were observed in ovarioles that also contained ≥1 tumorous follicle, so they were included in the counts for that category.

2 Observed adjacent to a follicle containing >16 germ cells.

3 Observed in ovarioles without follicles containing >16 germ cells, therefore included in the category ‘others’ in the upper section.

4 Phenotypes observed at very low penetrance (<1%) include binuclear germ cells, multilayered follicle cells, or unusually long stalks.

5 This phenotype was only scored in 54/173 ovaries.

We furthermore noted a number of additional phenotypes, mostly in ovarioles that contained at least one follicle containing more than 16 germ cells (to which we refer in the remaining text as ‘aberrant follicles’) (Table 1; Fig. 1). Three phenotypes were particularly common, and their frequencies are provided (Table 1). In many *cul-5^EY21463^* ovarioles containing follicles with more than 16 germ cells we also observed follicles with fewer than 16 germ line cells (Fig. 1F; Table 1). Many other aberrant *cul-5^EY21463^* ovarioles contained follicles with more than 16 germ cells that were either partially or fully split by a layer of somatic cells that extended through the germ cell area (Fig. 1I; Table 1; and data not shown). Notably, the bridge was not necessarily perpendicular to the long axis of the ovariole, and was sometimes oriented at odd angles or in parallel to the anterior-posterior axis of the follicle (not shown). The third prominent phenotypic category were ovarioles with ‘empty germaria’ (Fig. 1J; Table 1), in which the somatic cells seemed normally organized but no germ cells were detectable by α-Vas or α-Aub immunostaining. The phenotypic categories described in this paragraph are not mutually exclusive, and therefore a given ovariole may for instance display aberrant follicles, follicles with a somatic split and an empty germarium.

We next decided to establish whether the penetrance of these phenotypes increased with the age of the flies (Table 1). The aberrant follicle phenotype did not become fully penetrant even in flies that were 21–22d old. However, in flies aged for more than 10d, ovarioles were observed that were constituted of an disorganized array of germ cells of different sizes intermingled with somatic cells (Fig. 1K, L). We termed these aberrant ovarioles as having undergone ‘morphogenetic catastrophe’. Notably, the nuclear morphology of both somatic and germ line cells within ovarioles having suffered morphogenetic catastrophe was not consistent with pycnosis.

### Germ Cells in Aberrant Follicles Differentiate

**Figure 2:**
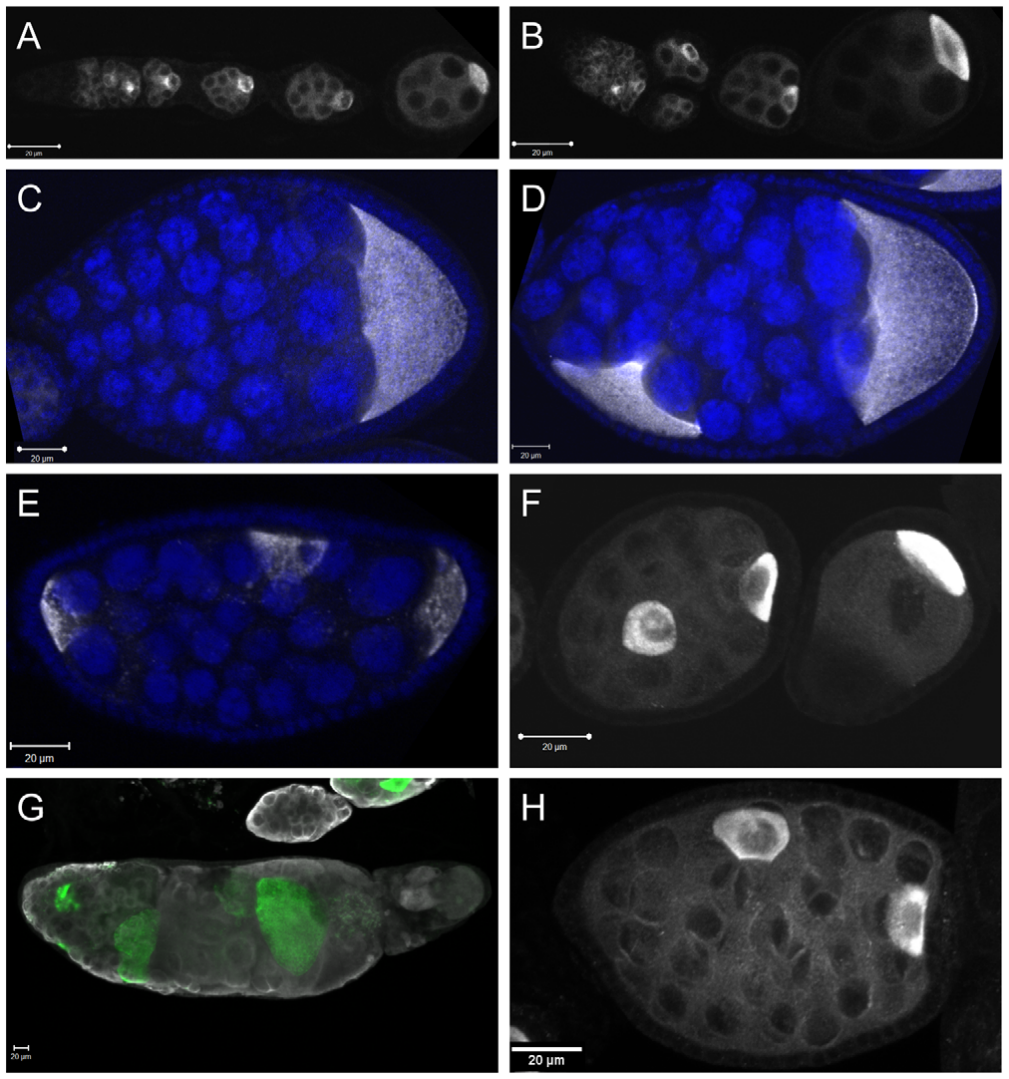
Germ cells in aberrant egg chambers differentiate into oocytes and nurse cells. Ovarioles and egg chambers from wild type (A) and *cul-5^ EY21463^* mutant (B–H) females. (A, B) Wild type and many *cul-5* mutant egg chambers accumulate the oocyte marker Orb (white) in a single cell in the posterior of the follicle. (C–E) While some aberrant egg chambers containing more than 16 germ cells contain only a single Orb-positive cell (white; DNA labelled blue), others contain two (D) or more (E) Orb-positive cells. (F) Follicles with fewer than 16 germ line cells may also contain an Orb-positive cell. (G) Ovarioles that have undergone morphogenetic catastrophe still contain Orb-positive cells of variable size (Orb is labelled green, Vas in white). (H) The oocyte marker Bic-D accumulates in a pattern very similar to Orb in *cul-5* mutant egg chambers.

We next asked whether the germ cells in the aberrant follicles take up nurse cell or oocyte identities and therefore maintain the capability to differentiate. We noticed that the majority of the Vas-positive cells contained large, obviously polyploid nuclei (Fig. 1, 2) that closely resemble wild type nurse cells, indicating that these cells have the capability of taking up a nurse cell fate. We then assayed whether all or some the germ cells within the aberrant follicles adopt an oocyte or oocyte-like fate. The germ cell-specific polyadenylation element-binding protein Orb is an early marker for oocyte fate in the germarium and remains highly enriched and asymmetrically distributed within the oocyte throughout oogenesis [15]. In *cul-5^EY21463^* ovarioles that did not contain aberrant egg chambers, Orb accumulated in a pattern that was indistinguishable from wild type (Fig. 2A, B). In many aberrant follicles Orb also was restricted to only one germ cell, usually located in the posterior of the egg chamber (Fig. 2C). This posterior Orb-positive cell often resembled a normal oocyte in size, shape and nuclear morphology; the former characteristics being most obvious in late stage follicles (Fig. 2C, D). However, many aberrant follicles contained more than one Orb-positive cell, in more or less random (but usually cortical) positions within the germ cell cluster (Fig. 2D-F). Notably, in most follicles that contained fewer than 16 germ cells, we also detected an Orb-positive cell (Fig. 2F), usually located at the posterior. Aged ovarioles that had undergone morphogenetic catastrophe also contained Orb-positive cells, scattered throughout and of significantly different sizes (Fig. 2G). To confirm these results, we stained *cul-5^EY21463^* ovarioles for Bic-D, another oocyte marker [16]. We also found Bic-D to be restricted to one or few germ cells in aberrant follicles, further supporting that some of these cells can adopt an oocyte fate (Fig. 2H; and data not shown). In conclusion, our observations suggest that germ cells within aberrant egg chambers continue to follow the developmental programs of the nurse cell and oocyte lineage and retain the ability to differentiate.

### Overproliferation in *cul-5^EY21463^* Germaria

**Figure 3:**
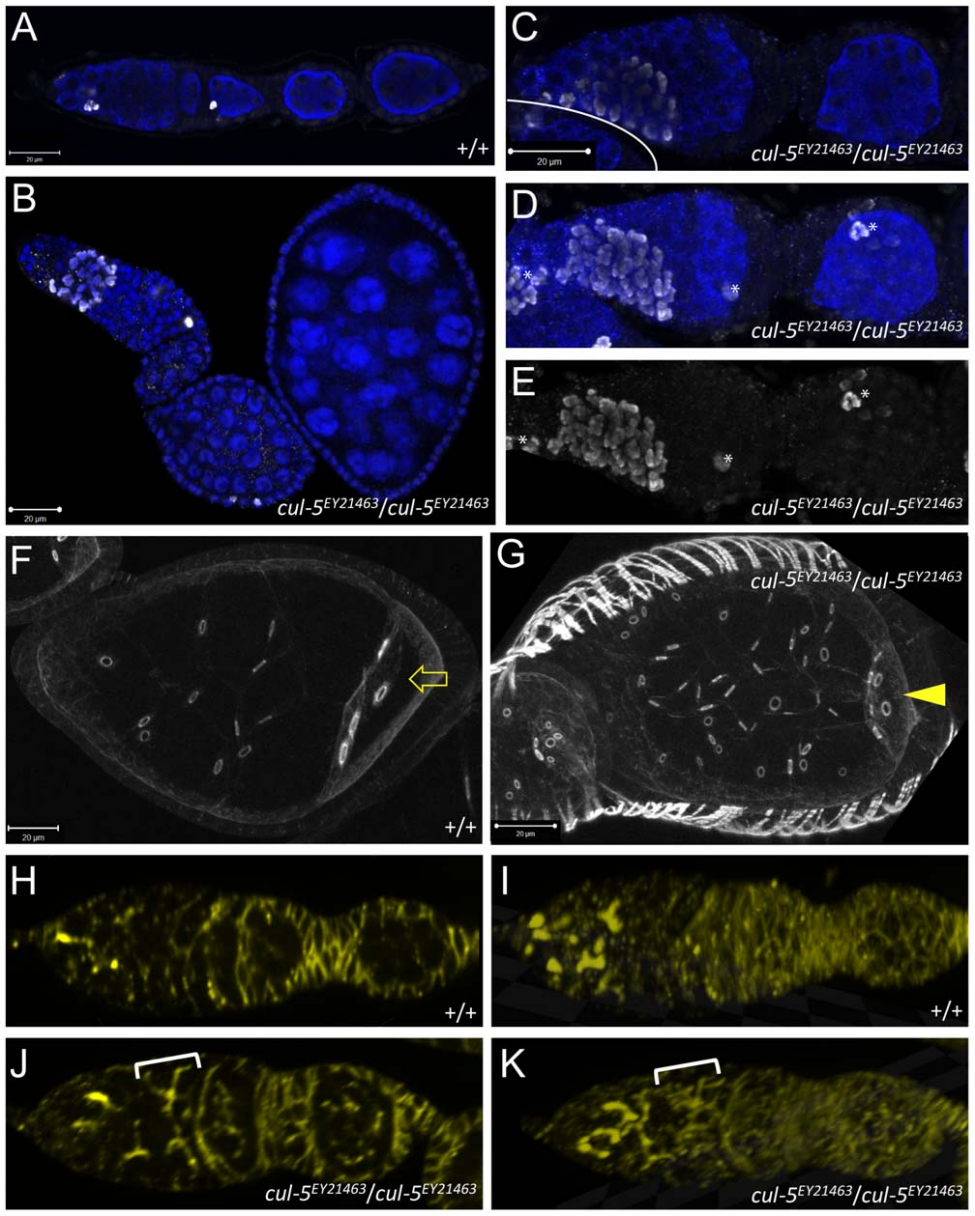
Germ line clusters overproliferate in the germarium of *cul-5* mutant ovarioles. (A–E) Wild type (A) and *cul-5^ EY21463^* mutant ovarioles (B–E) were stained for Vas (blue) and phospho-histone H3 (white). (A) In wild type ovarioles, small numbers of germ cells divide synchronously in the germarium. (B–E) In some *cul-5* mutant ovarioles, large numbers of germ line cells undergo mitosis in a germarium, while we never observe germ cell divisions in the distal germarium or in the vitellarium (B). (D–E) are projections of a Z-series taken through (C). The asterisk indicates dividing cells of somatic origin in the projections. (F) In wild type egg chambers, each oocyte (arrow) contains exactly four ring canals enriched in F-actin (labelled with Phalloidin). (G) In aberrant egg chambers in *cul-5* mutants, more than four ring canals are observed in oocytes (arrow) and nurse cells. (H–K) *cul-5* mutant germaria (J–K) feature large fusomes (labelled with mAb 1b1) spanning more than 16 cyst cells that are never observed in wild type (H–I) germaria. (H, J) are single 0.5 µm planes, while (I, K) are 3D reconstructions of corresponding Z-stacks spanning the width of the germaria. The bracket in K indicates a single polyfusome that is traceable through the entire Z-stack.

The observation that the aberrant follicles in *cul-5* mutant ovarioles contain excess germ cells prompted us to investigate whether this phenotype is due to germ line overproliferation. We used an antibody that recognises a phosphorylated form of Histone H3 (pH 3) characteristic of mitotic chromatin [17] to analyse germ cell proliferation in wild type and *cul-5* mutant ovarioles. As expected, we observed small numbers of pH 3-positive germ cells only in regions 1 and 2A in wild type germaria, and never in germ cells in more distal regions of the ovariole (Fig. 3A; and data not shown). Most *cul-5^EY21463^* germaria resembled wild type in this respect (not shown), but in some *cul-5^EY21463^* germaria we observed large clusters of synchronously dividing cells (Fig. 3B–E). We never observed pH 3-positive cells distal of the germarium (Fig. 3B; and data not shown).

It is technically difficult to unambiguously establish whether these synchronously dividing germ cells truly belong to a single cluster, or whether individual cyst cells within a cluster might divide asynchronously. However, two additional lines of evidence support the idea that some *cul-5* mutant germ cell clusters undergoes more than four rounds of synchronous mitotic divisions. Firstly, germ cells in cysts that have undergone the usual four rounds of incomplete cell division can have at most four ring canals [9], [10] (Fig. 3F). We frequently observe germ cells with five or six ring canals in aberrant *cul-5^EY21463^* follicles (Fig. 3G). We conclude that these cells have undergone more than four rounds of division. An alternative explanation, that membrane reductions causing fusions of cyst cells [18] underlie this phenotype is unlikely in this case, as we only extremely rarely observed multinuclear cells in *cul-5* mutant egg chambers. Therefore, syncytial cysts derived from more than four rounds of incomplete cell divisions are the more parsimonious explanation.

Secondly, we have observed branched polyfusomes in *cul-5^EY21463^* mutant germaria that clearly span more than 16 cells. Fusomes are ER-related structures that are essential to maintain both synchrony and asymmetry of germ line cyst divisions [12], [19]. During cyst division, fusomes undergo dynamic changes in morphology, however, in the mature 16-cell cyst, a single fusome temporarily extends through the ring canals of all cells of the cyst [20] (Fig. 3H). In *cul-5^EY21463^* mutant germaria, we detected fusomes that span more than 16 cells (Fig. 3I). This further implies that at least some germ cell cysts in *cul-5* mutant ovaries arise from more than four incomplete cell divisions.

### Aberrant Follicles Mostly Do Not Contain 2^n^ Germ Cells

**Figure 4:**
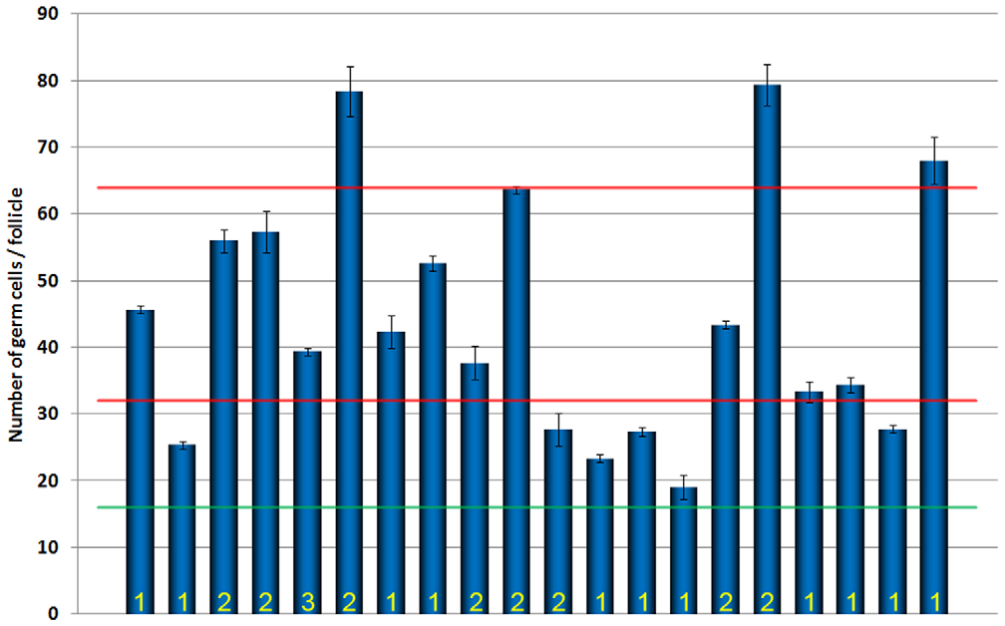
*cul-5* mutant follicles do not contain 2^n^ germ line cells. The graph plots the number of germ cells (Y-axis) in 20 randomly chosen aberrant follicles (X-axis). The green and red lines indicate the normal 16 (green) germ cells per follicles, or multiples of 16 (red). For each follicle, we counted the number of germ line cells three times (error bars denote ±SD). The number at the bottom of the column indicates the number of Orb-positive cells (oocytes) found in that particular follicle.

The synchronicity of cystocyte divisions necessitates that at the end of the proliferative period each cyst must contain 2^n^ cells [21], [22]. If extra cell divisions were the only cause for the formation of aberrant follicles with more than 16 germ cells, it would be expected that these would also follow the 2^n^ rule. To test this hypothesis, we counted the number of germ cells in 20 randomly chosen aberrant follicles in *cul-5^EY21463^* ovarioles (Fig. 4). Few aberrant follicles contained 32 or 64 germ cells, and most contained ‘odd numbers’. There are two possible explanations for this result. Firstly, germ cell cysts at some point could start to divide asynchronously, therefore making the final number of germ cells in a follicle unpredictable. We cannot fully rule out this possibility, although we did not observe small clusters or individually dividing cells in distal regions of the germarial region where germ cells divide, nor in the vitellarium, which would support it. Alternatively, encapsulation of the germ line cells by the follicular epithelium could be defective, leading to the formation of follicles containing several cysts enclosed together; in this process, individual cysts may also be split [11].

### Defective Cyst Encapsulation Contributes to the Formation of Aberrant Egg Chambers

During wild type oogenesis, the shape of the germ line cyst undergoes well-defined stereotypic shape changes in the germarium. In the most distal region of the germarium, cysts encapsulated by follicle assume first an oval and then a round shape as they bud off to enter the vitellarium [23] (Fig. 1A, B). In this region of many *cul-5^EY21463^* germaria, however, we noted large aggregates of germ line cells that took up ill-defined and variable shapes (Fig. 1C, D). Clearly, in these germaria more than the usual 16 cyst cells become encapsulated in, and bud off as, an aberrant follicle. The fact that we observe excessive germ cell numbers in the aberrant follicles already when leaving the germarium is consistent with our observation that germ cells do not divide in the vitellarium in *cul-5* mutant ovarioles. In some cases, somatic cells invade the population of germ cells in region 3 of the germarium (Fig. 1D), consistent with the idea that aberrant migration of follicle cells could lead to encapsulation of several germ line cysts in one follicle, as well as split an individual cyst into two parts. It is easy to envision that cysts containing more than 16 cells would be more prone to being split by invading follicle cells. Moreover, germ line cysts being split by invading somatic cells could very well account for the presence of follicles with fewer than 16 germ cells. Therefore, it appears reasonable to propose that both germ line overproliferation as well as improper morphogenesis of the follicular epithelium contribute to the formation of aberrant follicles in *cul-5* mutant ovaries.

To further investigate this possibility, we analysed the development of the follicular epithelium using lineage-specific markers. In the germarium, follicle cells either differentiate into main body follicle cells which cover most of the egg chamber, or they become prepolar cells (reviewed in [11]). Prepolar cells give rise to polar cells, which are situated at the anterior and posterior poles of the egg chamber, or to stalk cells which separate egg chambers from each other. Polar cell differentiation is essential for stalk cell formation, and for proper egg chamber encapsulation.

**Figure 5:**
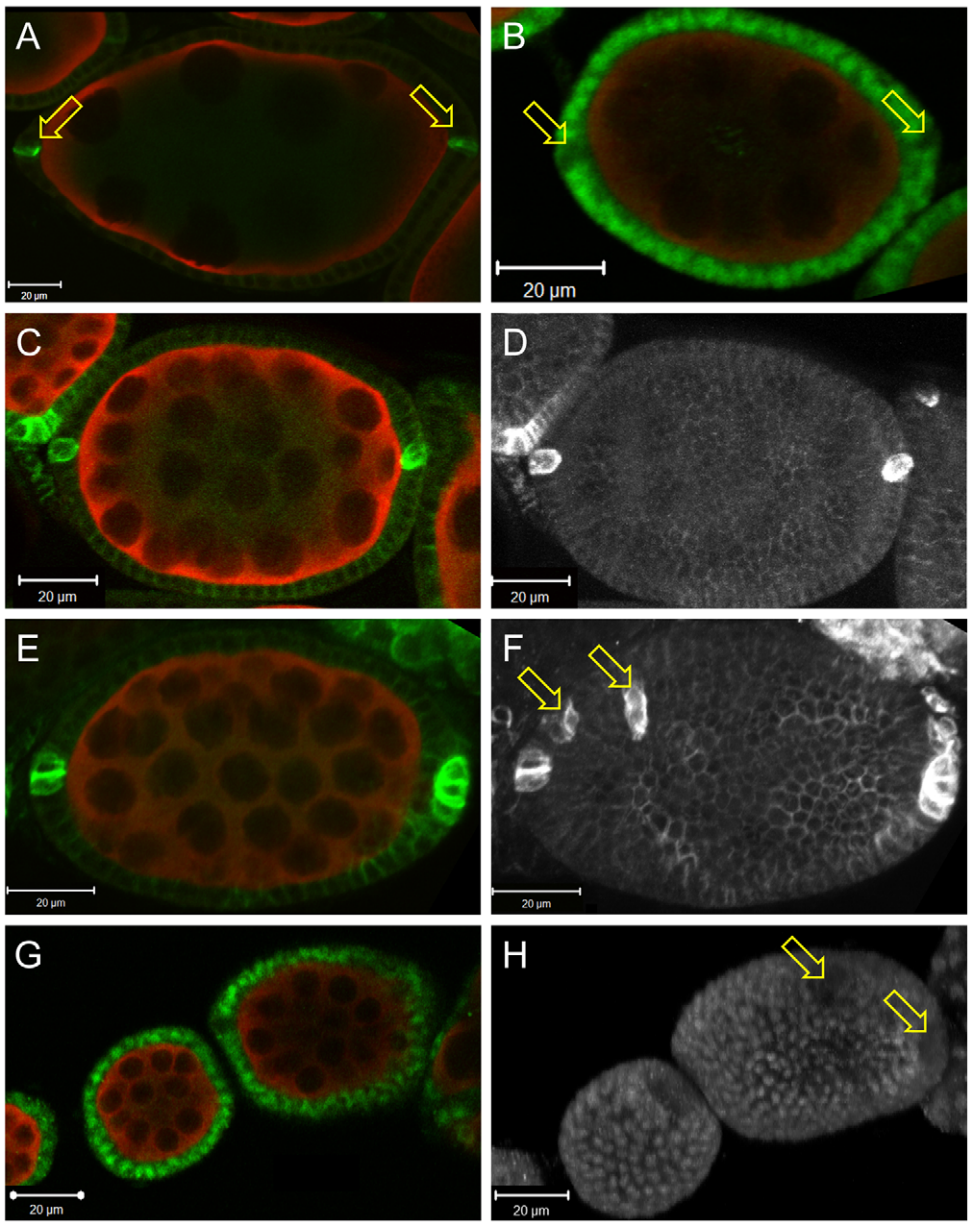
Follicular morphogenesis in aberrant *cul-5* mutant egg chambers. (A–H) Wild type (A–B) and *cul-5^ EY21463^* mutant (C–H) egg chambers were labelled for Vas (red) and FasIII (A, C–F) or EyA (B, G–H) (green in A–C, E, G or white in D, F, H). (A–C, E, G) are single confocal planes, while (D, F, H) are projections of Z-stacks spanning the entire corresponding egg chamber. (A) In wild type, FasIII accumulates in two pairs of polar cells at the anterior and posterior pole of the follicle (arrows). (B) EyA accumulates in a complementary pattern, and is excluded from the anterior and posterior polar cells while it accumulates in the nuclei of all other follicle cells. (C–D) In most aberrant egg chambers in *cul-5* mutants, only the normal two groups of polar cells are specified. (E–H) Only in small fraction of aberrant *cul-5* follicles, more than two groups of cells have upregulated FasIII (E–F) or downregulated EyA (G–H).

We analysed wild type and *cul-5^EY21463^* mutant ovaries with two markers that distinguish these different sub-populations of follicle cells. Firstly, we used an antibody that recognizes the homophilic cell adhesion molecule Fasciclin III (FasIII) [24]. In very young wild type egg chambers, FasIII is highly enriched in the membranes of all follicle cells but then becomes restricted to the polar cells, which ultimately form two two-cell clusters at the anterior and posterior poles of the follicle [11], [25] (Fig. 5A). Secondly, we utilized anti-EyA, which recognizes Eyes absent protein (EyA), a DNA-associated phosphatase that represses polar cell fate and is enriched in all follicle cells except the polar cells [11], [26] (Fig. 5B). In the majority of *cul-5^EY21463^* follicles, aberrant or not, both staining patterns resembled wild type in that FasIII was enriched in an anterior and a posterior population of polar cells but downregulated in all other follicle cells, while EyA was detectable in its complementary pattern (Fig. 5C, D; and data not shown). However, in 8.0% of the aberrant follicles (n = 75) we detected more than two populations of FasIII-positive cells (Fig. 5E, F). Our experiments with anti-EyA confirmed our FasIII results; in most egg chambers we only observed two populations with downregulated EyA (not shown) while we detected a small fraction of aberrant follicles that contained additional groups of cells lacking detectable EyA expression (Fig. 5G, H). The EyA-negative cells most likely correspond to the supernumerary FasIII-positive clusters. The downregulation of EyA in these cells argues that they are differentiated polar cells, not merely undifferentiated prefollicular cells.

Taken together, these and the results described above support a model in which germ cell overproliferation and defects in the morphogenesis of the follicular epithelium both contribute to the formation of aberrant follicles.

### Reduced *gus* Function Drastically Enhances the *cul-5* Phenotype

The substrate specificity receptor Gus has been co-crystallized with Vas [27], [28]. Gus contains a SOCS-box domain that is predicted to interact with the Elongin BC complex to recruit Cul-5 containing ubiquitin E3 ligases to proteins, such as Vas, with which it interacts. Consequently, we asked whether altering *gus* activity might affect the *cul-5* phenotypes we observed. We never observed aberrant egg chambers in *cul-5^EY21463^*;*gus^f070073^* transheterozygotes (Table 2) or in *gus^f07073^* homozygotes (data not shown), or in *gus^f07073^* homozygotes that bear only one copy of *cul-5^EY21463^* (Table 2). However, 82% of *gus^f07073^*/+;*cul-5^EY21463^*/*cul-5^EY21463^* ovarioles contained one or more aberrant egg chambers (Table 2), as compared to only 34% in *cul-5^EY21463^* ovaries (Table 1, 2). We also saw increased penetrance of *cul-5* mutant phenotypes in *gus^f07073^*;*cul-5^EY21463^* double homozygous females. Almost all ovarioles of this genotype contained aberrant follicles, and those not containing clearly identifiable aberrant follicles had undergone morphogenetic catastrophe, even when only 1–2d old. Notably, most of the cells in these malformed *gus;cul-5* ovarioles had pycnotic nuclei. This clear enhancement of the *cul-5* mutant phenotype by *gus* mutations strongly supports the idea that their products operate in a common pathway to ensure appropriate follicular morphogenesis. Intriguingly, we observed a high proportion (52.9%) of empty germaria in *gus^f07073^*;*cul-5^EY21463^* ovaries. This suggests that a Gus-containing Cul-5 CRL is involved in the maintenance of germ line cells in the ovariole.

**Table 2:** *gus* mutations enhance *cul-5* phenotypes.

	*gus/+; cul-5/+*	*gus/gus; cul-5/+*	*cul-5/ cul-5*	*gus/+; cul-5/cul-5*	*gus/gus; cul-5/cul-5*
n	150	150	105	172	87
% no phenotype	100.0	100.0	65.7	16.3	0.0
% tumorous	0.0	0.0	34.3	82.0	82.8
% morphogenetic catastrophe	0.0	0.0	0.0	0.0	17.2
% others	0.0	0.0	0.0	1.7	0.0
% empty germarium	0.0	0.0	7.6	14.5	52.9
% somatic tunnel	0.0	0.0	3.8	16.3	9.2
% <16 germ cells	0.0	0.0	2.9	9.3	11.5

## Discussion

We present evidence that Cul-5 is important for normal follicular development in *Drosophila* oogenesis. We find that reduced *cul-5* activity predominantly causes formation of aberrant egg chambers that contain abnormal numbers of germ cells. Our data suggest that both abnormal proliferation of germ cell cysts as well as aberrant packaging of germ cell cysts into follicles contribute to this phenotype. Although few direct targets of *Drosophila* Cul-5 have been identified, it seems likely that Cul-5-containing CRLs would modify many proteins. Therefore, it would be unsurprising if several pathways acting in follicular morphogenesis were affected by reduced *cul-5* function.

### Abnormal Morphogenesis of the Follicular Epithelium Contributes to the Formation of Aberrant Follicles

Aberrant egg chambers containing more or less than the normal complement of 16 germ line cells may arise for a number of different reasons [11]. Several reports link Epidermal Growth Factor Receptor (EGFR) signalling to proper follicle formation. EGFR, together with the neurogenic genes *egghead* and *brainiac*, is required for normal egg chamber separation [29], [30], [31], [32]. EGFR is a particularly interesting putative target of Cul-5-dependent regulation, because EGFR and Gus interacted in a global yeast-two-hybrid screen [33], and EGFR therefore may be directly regulated by Cul-5/Gus CRLs. It is noteworthy, however, that Gus appears to be predominantly expressed in germ line cells in the germarium, at least as judged by immunocytochemistry [13]. In apparent contrast, EGFR is required in somatic cells for proper ovarian tissue morphogenesis. It remains to be established whether EGFR is a bona fide interactor of Gus, and whether small amounts of Gus expressed in somatic cells of the germarium may regulate EGFR. Alternatively, germ line-expressed Gus could regulate EGFR indirectly, for instance by modulating the expression, secretion or activity of one or several EGFR ligands.

EGFR appears to affect migratory movements of early follicle cells to surround each germ line cyst, because *Egfr* mutant follicle cells fail to correctly migrate between individual cysts [30], [31]. As a consequence, multiple cysts become enclosed in a single follicular epithelium, leading to the formation of aberrant egg chambers. One possible explanation for this phenomenon is that EGFR signalling affects adhesion between follicle and germ line cells [31]. This model is attractive, as for instance Cadherins are required for follicular morphogenesis [34], [35]. Moreover, loss of β-PS integrin from follicle cells can lead to failures in egg chamber encapsulation and cause germ line cyst splitting [36]. However, it is also possible that EGFR signalling is required for adhesion between different germ cells [32], which could explain how follicle cells can invade individual cysts and cause their splitting. Loss of Armadillo/β-catenin in germ line cells may cause formation of aberrant egg chambers [37]; however, it is not clear whether the mutant phenotype is due to a loss of a cell adhesion or because of a signalling function of β-catenin. Therefore, it seems possible that Cul-5 CRLs impact on cell adhesion properties through an effect on EGFR signalling, or on downstream cell adhesion molecules or their regulators.

EGFR is not the only signalling pathway implicated in follicle encapsulation. Mutations affecting Notch, Delta or the Notch modulator Fringe can cause formation of large compound egg chambers which contain several individual cysts, or can prevent the formation of stalk cells leading to fused egg chambers separated by a follicular epithelium but not a stalk [25], [38], [39], [40]. These results imply that Notch signalling is required to specify polar cells which are in turn essential for stalk cell formation, therefore reduced Notch activity causes fusion of egg chambers. Hedgehog and Dpp signalling also have been implicated in proper egg chamber formation [11].

### Germ Cell Overproliferation Contributes to the Formation of Aberrant Egg Chambers

While many aspects of the *cul-5* phenotype resemble the effects of mutations in the EGFR and Notch/Delta pathways, the germ cell overproliferation phenotype of *cul-5* is not exhibited by mutants in these signaling pathways. Overexpression of Cyclin A, or mutations affecting the cyclin-dependent kinase inhibitor Dacapo or the E2 conjugating enzyme UbcD1 may cause one extra round of cyst divisions, leading to the production of follicles containing 32 germ cells [41], [42]. These egg chambers generally contain only a single oocyte with five ring canals and always follow the 2^n^ rule, and abnormal morphogenesis of the follicular epithelium does not appear to contribute to the generation of aberrant egg chambers in these cases. Mutations in *encore* also cause extra germ line cyst divisions, and *encore* egg chambers are occasionally split, leading to the formation of follicles with fewer than 16 germ line cells [43]. This indicates that Encore may also be involved in follicular morphogenesis; however, the small *encore* follicles do not specify an oocyte, unlike what we observed for *cul-5*. Encore has been shown to associate with Cul-1 CRLs and to regulate Cyclin E expression in the germarium [44]. Therefore, Cul-5 and Encore have in common that they act in ubiquitin-dependent pathways that affect both germ line cyst divisions as well as morphogenesis of the follicular epithelium.

Large scale yeast two hybrid screens [33], [45] provide interesting candidates for substrates of Cul-5/Gus CRLs that are related to cell cycle control. For instance, Gus was shown to interact with the Cyclins CycA, CycG, CycH and CycJ, as well as with the Cyclin-dependent kinases CDK2 and CDK4, and with the transcription factor E2f2. While direct regulation of any of these putative targets through a Gus-Cul-5 CRL remains to be shown, it is attractive to speculate that the germ line overproliferation phenotype of *cul-5* mutants may be due to aberrant regulation of one or several regulators of cell proliferation. We never observed germ cell overproliferation in *gus* mutants (data not shown), however, since no null allele of *gus* is currently available we cannot distinguish whether the overproliferation phenotype occurs exclusively because of reduced Cul-5/Gus CRL activity or whether Cul-5 CRLs using substrate receptors other than Gus are involved in cell cycle regulation. It would be highly informative to generate and analyse null alleles for both *gus* and *cul-5* to address this and other questions.

### A Cul-5/Gus CRL Is Likely Involved in Germ Line Stem Cell Maintenance or Germ Cell Survival

We observed germaria devoid of germ cells in *cul-5* mutant ovarioles, which indicates either defects in GSC maintenance or induction of ectopic germ cell death. We occasionally observed germaria which were devoid of GSCs but still contained one or very few clusters of multicellular germ line cysts (data not shown; of note, this phenotypic category is not included in the ‘empty germaria’ category in our scoring scheme). This observation is consistent with the idea that GSCs may differentiate inappropriately in *cul-5* mutants resulting in their loss, leading to the empty germaria phenotype. In contrast, we never observed obviously aberrant nuclear morphology in the germarial germ cell population by DAPI stainings. However, based on our data we cannot fully rule out that reduced germ cell or GSC survival causes or contributes to the germ line depletion phenotype in *cul-5* mutant ovaries. Interestingly, this phenotype is strongly enhanced in *gus; cul-5* double mutants, suggesting that Cul-5/Gus CRL act to prevent GSC differentiation or to ensure their survival. Existing *gus* mutations do not display such a phenotype (data not shown), therefore it remains possible that Cul-5 CRLs other than Gus are also involved in germ line maintenance.

### Conclusion

We provide evidence that Cul-5 CRLs are involved in *Drosophila* follicular morphogenesis, in the regulation of germ line cyst divisions and in germ line maintenance, and that some of these Cul-5 CRLs most likely include Gus. Our results suggest that Cul-5 regulates numerous targets relevant to these phenotypes. Identification of these Cul-5 targets will require further investigation using biochemical and proteomic approaches.

## Materials and Methods

### Fly Stocks

The *cul-5^EY21463^* and *cul-5^EY00051^* alleles were obtained from the Bloomington Stock Center.

### Immunostainings and Microscopy

0–24 h old flies were collected and fed on dry yeast for 24 h prior to dissection. To obtain aged specimens, 0–24 h old flies were collected and maintained on standard cornmeal-molasses food that was changed every 2–3 days. Ovaries were dissected into PBS, fixed in 4% formaldehyde in PBS for 5 minutes, rinsed 3x in PBST (PBS +0.3% Triton), washed extensively first in PBST, then in PBSTB (PBST +3% BSA) prior to incubation in primary antibodies diluted in PBSTB over night. Samples were then rinsed and washed extensively prior to overnight incubation in secondary antibodies diluted in PBSTB. Samples were then rinsed and washed in PBSTB, followed by 20 minutes incubation in 0.5 µg/ml DAPI in PBST. After one rinse in PSBT, samples were equilibrated in Slow Fade Antifade Reagent (1 part Antifade solution in 6 parts equilibrium buffer) before mounting. All steps were performed at room temperature, except for antibody incubations, which were done at 4°C. Rhodamine-Phalloidin incubation was performed together with the secondary antibodies when applicable.

Primary antibodies used were: rabbit α-Vas (1∶2000), rabbit α-Aub (1∶2000), mouse α-Orb (DSHB clones 4H8 and 6H4, used together at 1∶30 each), mouse α-Bic-D (DSHB clone 1B11, 1∶20), rabbit α-PH 3 (Upstate, 1∶500), mouse α-adducin-related protein (DSHB clone 1B1, 1∶15), mouse α-FasIII (DSHB clone 7G10, 1∶50) and α-EyA (DSHB clone eya10H6, 1∶50). Secondary antibodies were AlexaFluor-488- and AlexaFluor-555-conjugated (Molecular Probes) and used at 1∶500.

Samples were analysed either on a Leica DM6000B fluorescence microscope, a Zeiss LSM510meta confocal microscope, or a Leica DMI6000B spinning disc confocal microscope. Image acquisition was done with Volocity or LSM imaging software. For image analysis and figure preparation we used the LSM Image Browser, Volocity, ImageJ and Adobe Photoshop and Illustrator.
